# Cigarette access and purchase patterns among adolescent smokers aged 12-16 years in 140 countries/territories, Global Youth Tobacco Survey 2010-2018

**DOI:** 10.7189/jogh.12.04101

**Published:** 2022-12-21

**Authors:** Jiahong Sun, Bo Xi, Chuanwei Ma, Zilin Li, Min Zhao, Pascal Bovet

**Affiliations:** 1 Department of Epidemiology, School of Public Health, Qilu Hospital, Cheeloo College of Medicine, Shandong University, Jinan, Shandong, China; 2 Department of Nutrition and Food Hygiene, School of Public Health, Cheeloo College of Medicine, Shandong University, Jinan, Shandong, China; 3 Center for Primary Care and Public Health (Unisanté), University of Lausanne, Lausanne, Switzerland

## Abstract

**Background:**

Few studies have examined access to cigarettes among adolescents. To address this, we aimed to examine cigarette access and purchase patterns among adolescent cigarette smokers based on the most recent data from the Global Youth Tobacco Surveys (GYTS).

**Methods:**

We used the most recent school-based GYTS data (2010-2018) on 49 856 adolescent cigarette smokers aged 12-16 years in 140 countries/territories (hereafter “countries”) to examine cigarette access and purchase patterns among adolescent smokers.

**Results:**

Over half (51.2%) of adolescent smokers bought cigarettes from commercial vendors (ie, stores/shops/street vendors/kiosks) and one-third of smokers (32.7%) got cigarettes from private persons (ie, peers or adults). Males (vs females), older adolescents (vs younger ones), and adolescent smokers from low-income countries (vs those from middle- or high-income countries) were more likely to buy cigarettes from commercial vendors. Younger adolescents (vs older ones) and adolescent smokers from low-income countries (vs those from middle- or high-income countries) were more likely to get cigarettes from private persons. As many as 39.6% of adolescent smokers reported that they were not denied buying cigarettes from commercial vendors due to age restrictions, especially among males (vs females), among older adolescents (vs younger ones), and among adolescent smokers from upper-middle-income or high-income countries (vs those from low-income countries). Purchasing cigarettes as single sticks was more likely to occur in males (vs females), in older adolescents (vs younger ones), and in adolescent smokers from low-income countries (vs those from upper-middle-income or high-income countries), with inverse findings for purchasing cigarettes in full packs.

**Conclusions:**

Adolescent smokers often obtained cigarettes from commercial vendors or private persons. Additionally, adolescent smokers often purchased cigarettes in packs and as individual sticks. These findings emphasize the need to strengthen measures to reduce the supply of cigarettes to minors.

Tobacco use is a leading preventable cause of morbidity and mortality [1,2], with nearly eight million deaths attributable to tobacco use worldwide and a total economic cost of the tobacco-related burden of around US$1.4 trillion per year [3]. The World Health Organization (WHO) estimates that 80% of the 1.3 billion people using tobacco products are located in low- and middle-income countries, which are prime targets of the tobacco industry, often because of loose tobacco control regulations in such countries [3]. As estimated in 2019, 155 million young people aged 15-24 years used a tobacco product worldwide, emphasizing the unique window of opportunity for tobacco control [4].

Tobacco use usually starts in childhood or adolescence and tracks into adulthood due to the addictive nature of nicotine [5]. A recent study of 530 234 adolescents aged 13-15 years from 143 countries/territories (hereafter “countries”) based on the Global Youth Tobacco Surveys (GYTS) in 2010-2018 showed that the global prevalence of tobacco use (on at least one day during the past 30 days) exceeded 10% [6]. This is partly due to aggressive cigarette marketing (eg, via cigarette displays in stores or the internet) and the lack of enforcement of regulations restricting cigarette sales to minors [7]. Some supply-side measures that can reduce tobacco use by adolescents are reducing access to tobacco products by minors by enforcing age checking by cigarette retailers, raising the legal age for tobacco sales, and banning cigarette sales by units [8,9]. However, limiting the supply of cigarettes to minors does not necessarily prevent adolescents from getting cigarettes from private individuals, including friends and other social contacts [10-12].

Only three studies have examined how adolescents obtain cigarettes in different regions [13-15]. One previous study in 45 countries based on GYTS in 2013-2014 showed that the proportion of adolescent smokers purchasing cigarettes from commercial vendors (eg, stores, shops, street vendors, or kiosks) exceeded 50% in many countries (26 of 45 countries) [13]. Another study based on GYTS data in 2009-2011 in six sub-Saharan African countries showed that over 20% of adolescent smokers bought cigarettes from commercial vendors [14]. A study in 2016-2017 in seven European countries showed a similar pattern in half of the included countries examined [15]. Additionally, GYTS (2013-2014) showed that adolescent smokers purchased cigarettes as individual sticks in most countries in the African and South-East Asian regions, and they tended to purchase cigarettes in packs (vs sticks) among most countries in the European region [13]. However, these data are either relatively old or based on a limited number of countries. Considering differences in population characteristics and socioeconomic factors across countries and the potential effect of cigarette access on future cigarette initiation [16] which may cause short-term and long-term adverse clinical outcomes [17], it is necessary to understand the status of cigarette access and purchase patterns among adolescents globally to guide the policy formulation and regulation establishment at national levels.

We used the most recent data 2010-2018 GYTS data from 140 countries to examine how adolescent smokers aged 12-16 years purchased cigarettes, particularly whether they got cigarettes from commercial vendors vs private persons, and in pack vs as individual sticks.

## METHODS

### Data source

We obtained data from the most recent GYTS (2010-2018) conducted in each included country among school-attending students aged 12-16 years. If a country conducted more than one survey between 2010 and 2018, we used only the latest one. The same sampling strategy and standardized questionnaire methodology allow for a direct comparison of data across countries and over time. A detailed description of the GYTS is available from the websites of the US Centers for Disease Control and Prevention (CDC) and WHO [18]. All GYTSs were approved by the ethics committee of each country.

### Questionnaire

One question assessed on how adolescents obtained cigarettes (“The last time you smoked cigarettes during the past 30 days, how did you get them?”), with responses including bought from stores/shops/supermarkets/gas station shops, street vendors/un-established vendors/kiosks, a vending machine, someone else (eg, peers, elders), or from other ways (eg, stealing) [19]. Another question assessed whether adolescents were denied purchasing cigarettes from commercial vendors because of age (“During the past 30 days, did anyone refuse to sell you cigarettes because of your age?”), with possible responses being yes or no [19]. A third question assessed whether adolescents bought cigarettes in packs or as individual sticks (“At the last time you bought cigarettes during the past 30 days, how did you buy them?”), with responses including bought in packs, as individual sticks, and other patterns (ie, in a carton, in rolls) [19]. The GYTS questions on tobacco purchase patterns have been demonstrated to have good test-retest reliability [20,21]. The income category of each country using the World Bank classification was based on data on the year when GYTS was last conducted.

### Statistical analysis

We used sampling weights in each GYTS survey to calculate the weighted proportion estimates and 95% confidence intervals (CIs) using the “Complex Samples” module in SPSS (version 16.0). Subgroup analyses were performed according to sex, age, WHO region, and World Bank income. We calculated pooled estimates in the total population and in population subgroups by meta-analyses using STATA version 11.0 with random-effects models due to high heterogeneity between countries and used the χ^2^ test to examine differences in proportions between groups. We used the “pwr” package in R to calculate the statistical power based on the sample size and proportion with α = 0.05. The statistical power was higher than 0.80 for most subgroups (Table S1 in the **Online Supplementary Document**). We used logistic regression analyses to examine the association of age, sex, and income with cigarette access and purchase patterns (separately for access to cigarettes from commercial vendors or private persons vs other sources, buying cigarettes in packs vs as individual sticks, and not being denied buying cigarettes vs denied buying cigarettes). *P* < 0.05 indicates a significant statistical difference.

## RESULTS

Table S2 in the **Online Supplementary Document** shows the characteristics of the GYTS surveys in different countries. Data were collected in 140 countries from the six WHO regions, including regions of Africa (22 countries), America (30 countries), Eastern Mediterranean (24 countries), Europe (32 countries), South-East Asia (nine countries), and Western Pacific (23 countries). The data included 49 856 adolescent cigarette smokers (males = 65.6%) from 509 541 adolescents aged 12-16 years.

As shown in **Table 1**, during the 30 days prior to the survey, 51.2% of adolescent smokers bought cigarettes from commercial vendors (stores/shops/street vendors/kiosks), 32.7% from someone else (ie, private persons), 14.2% from other ways (eg, stealing), and 3.1% from a vending machine. Males (vs females) and older adolescents (vs younger ones) were more likely to buy cigarettes from commercial vendors, whereas females (vs males) and younger adolescents (vs older ones) were more likely to get cigarettes from private persons and other sources. Older adolescents (vs younger ones) were more likely to get cigarettes from a vending machine. The proportions of adolescent smokers purchasing cigarettes from commercial vendors or private persons did not vary substantially across WHO regions, except for South-East Asia compared with America. The proportion of adolescents who got cigarettes from a vending machine was generally low (3.1%). Moreover, adolescent smokers obtained cigarettes more often from private persons, from a vending machine, and through other ways in high-income vs lower-middle-income countries, but they purchased cigarettes more often from commercial vendors in low- and middle-income vs high-income countries.

**Table 1:** Proportions (%) of adolescent smokers purchasing cigarettes from commercial vendors, private persons, vending machines, and other ways, and frequency of purchasing cigarettes in packs or as single sticks according to sex, age group, WHO region and World Bank income, 2010-2018

Group	Source of cigarette purchase*						No refusal of sale from commercial vendors due to age*	Purchase patterns*		
	**No of countries**	**No. of sample size**	**Bought in stores/shops/street vendors/kiosks**	**Got from private persons**	**Bought from vending machine**	**Other ways**		**Pack**	**Individual sticks**	**Other patterns**
**Total**	140	49 856	51.2 (47.7-54.7)	32.7 (29.2-36.2)	3.1 (2.5-3.8)	14.2 (12.8-15.6)	39.6 (37.4-41.9)	46.3 (42.1-50.6)	37.1 (33.1-41.0)	14.7 (13.4-16.1)
**Sex**										
Males	140	30 565	55.3 (51.5-59.1)	29.8 (26.5-33.1)	3.3 (2.5-4.1)	12.3 (11.1-13.5)	40.4 (37.8-43.0)	45.9 (41.8-50.1)	37.5 (33.3-41.8)	15.0 (13.5-16.6)
Females	140	19 291	43.3 (34.8-51.8)	39.2 (36.0-42.3)	3.5 (2.4-4.6)	16.1 (14.4-17.7)	37.3 (31.9-42.6)	48.6 (43.1-54.1)	35.1 (30.9-39.3)	13.0 (11.4-14.6)
*P*-value			0.006	<0.001	0.387	<0.001	0.154	0.221	0.216	0.039
**Age group**										
12-14 y	140	19 274	48.0 (43.7-52.3)	35.1 (30.8-39.4)	2.8 (2.1-3.5)	16.5 (14.9-18.2)	34.7 (32.1-37.4)	44.1 (39.9-48.4)	37.8 (33.6-42.1)	16.3 (14.5-18.2)
15-16 y	140	30 582	54.6 (50.9-58.2)	31.2 (27.9-34.5)	4.3 (2.8-5.8)	11.3 (10.1-12.4)	43.5 (40.5-46.5)	48.0 (43.1-52.9)	37.1 (32.7-41.4)	12.4 (11.1-13.8)
*P*-value			0.011	0.079	0.038	0.011	<0.001	0.119	0.411	<0.001
**WHO region**										
Africa	22	5067	53.8 (44.1-63.5)	29.0 (20.3-37.7)	7.8 (3.2-12.4)	14.4 (11.9-17.0)	37.5 (33.3-41.7)	27.9 (21.3-34.5)	48.5 (39.2-57.9)	20.4 (15.3-25.6)
America	30	7430	46.9 (39.7-54.0)	36.1 (26.8-45.4)	1.3 (0.9-1.7)	13.7 (11.1-16.3)	38.8 (35.3-42.2)	40.8 (35.0-46.7)	40.7 (34.9-46.4)	14.7 (11.9-17.5)
Eastern Mediterranean	24	3656	54.2 (46.7-61.7)	28.2 (21.7-34.7)	6.9 (1.7-12.0)	16.0 (11.7-20.2)	46.1 (42.3-49.8)	45.9 (38.0-53.8)	36.5 (28.1-44.9)	17.1 (13.5-20.6)
Europe	32	25 116	54.2 (46.6-61.8)	30.1 (26.9-33.3)	5.0 (2.8-7.1)	14.5 (11.3-17.6)	43.0 (35.9-50.1)	68.5 (64.0-73.0)	18.1 (14.0-22.3)	12.6 (10.6-14.6)
South-East Asia	9	2408	57.8 (48.0-67.5)	30.2 (24.3-36.2)	1.2 (0.0-2.6)	6.5 (3.7-9.3)	39.0 (33.3-44.6)	28.4 (20.0-36.9)	57.2 (39.3-75.2)	14.5 (7.0-22.0)
Western Pacific	23	6179	44.9 (34.9-54.9)	40.8 (30.2-51.4)	1.7 (0.8-2.7)	14.8 (11.3-18.3)	32.1 (28.0-36.3)	44.9 (35.4-54.4)	41.7 (31.7-51.7)	12.2 (8.8-15.6)
*P*-value (America as reference)†										
*Africa*			0.131	0.137	0.003	0.353	0.320	0.002	0.082	0.028
*Eastern Mediterranean*			0.084	0.086	0.017	0.183	0.002	0.166	0.209	0.149
*Europe*			0.085	0.116	<0.001	0.351	0.149	<0.001	<0.001	0.116
*South-East Asia*			0.039	0.147	0.443	<0.001	0.476	0.009	0.043	0.480
*Western Pacific*			0.375	0.257	0.223	0.310	0.007	0.236	0.433	0.133
**World Bank income**										
Low	18	2972	53.1 (41.3-65.0)	28.6 (19.7-37.5)	3.9 (1.5-6.2)	16.5 (11.0-22.0)	39.4 (34.6-44.2)	33.5 (21.8-45.3)	45.4 (33.5-57.3)	19.1 (13.8-24.4)
Lower middle	42	11 112	58.2 (51.0-65.5)	28.1 (22.1-34.2)	2.4 (1.6-3.3)	11.6 (9.4-13.7)	37.5 (35.1-40.0)	36.3 (30.5-42.0)	48.0 (40.6-55.3)	14.0 (11.4-16.7)
Upper middle	46	24 626	52.3 (47.6-56.9)	32.6 (26.2-39.0)	2.7 (1.4-4.1)	12.8 (11.0-14.7)	41.0 (36.3-45.7)	51.9 (45.5-58.3)	32.7 (26.9-38.4)	12.0 (10.4-13.7)
High	34	11 146	40.6 (33.4-47.8)	39.8 (35.2-44.4)	5.3 (2.9-7.9)	17.3 (14.0-20.5)	40.1 (34.3-45.9)	58.7 (53.0-64.4)	24.3 (19.0-29.6)	16.4 (13.8-19.1)
*P*-value (lower middle as reference)†										
*Low*			0.236	0.464	0.120	0.052	0.245	0.337	0.358	0.046
*Upper middle*			0.090	0.158	0.356	0.203	0.098	<0.001	0.001	0.105
*High*			<0.001	0.001	0.016	0.002	0.209	<0.001	<0.001	0.105

WHO – World Health Organization

*Data are presented as proportions (95% confidence intervals).

†America (with an intermediate proportion) and lower middle level (with an intermediate income) were used as references.

**Figure 1:**
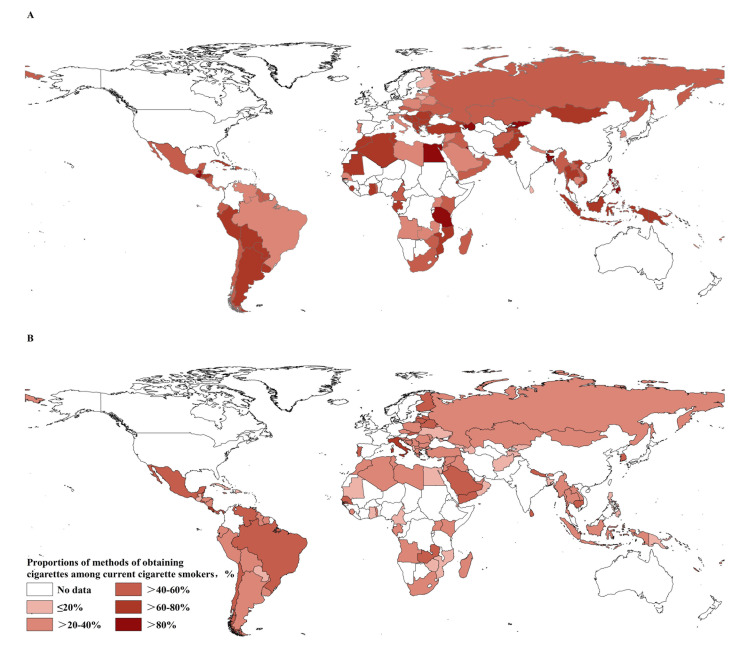
Proportions of adolescent smokers across countries who obtained cigarettes from **Panel A:** commercial vendors and **Panel B:** private persons.

The proportion of adolescent smokers purchasing cigarettes from commercial vendors varied largely across countries, ranging from 4.7% in Tokelau to 91.1% in the United Republic of Nevis (**Figure 1**, panel A, Table S3 in the **Online Supplementary Document**). The proportion of adolescent smokers purchasing cigarettes from private persons (**Figure 1**, panel B and Table S3 in the **Online Supplementary Document**) and a vending machine (Table S3 in the **Online Supplementary Document**) also varied largely across countries.

**Figure 2:**
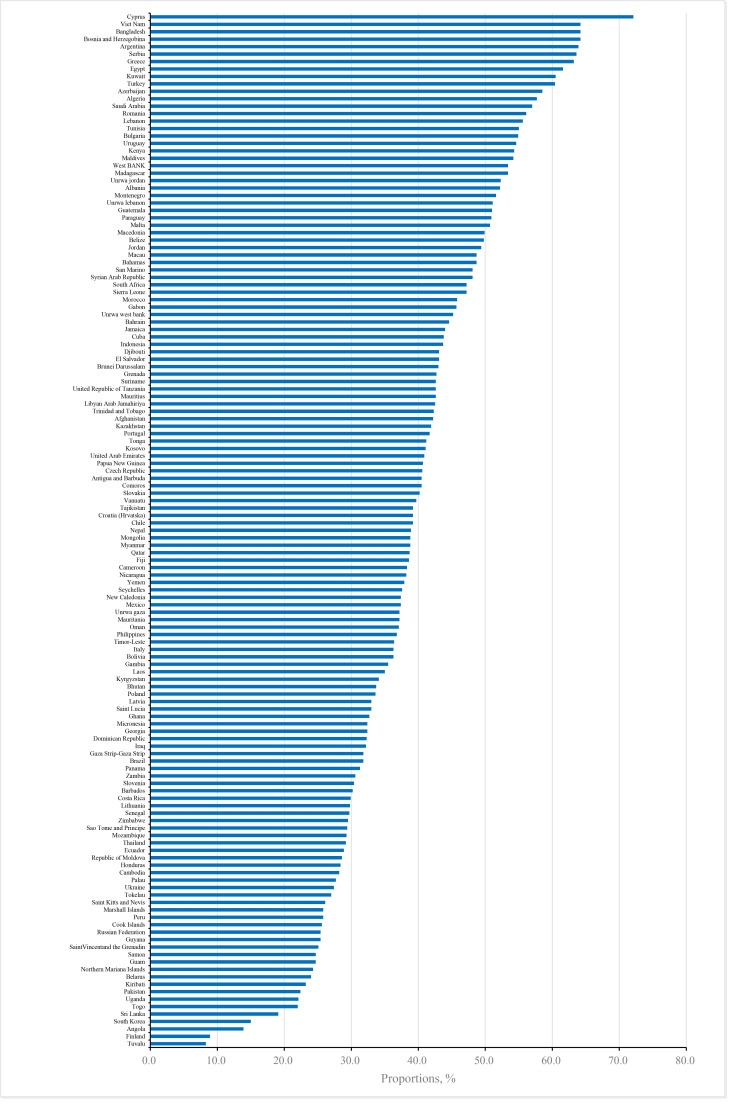
Proportion of adolescent smokers not denied sale of cigarettes by commercial vendors.

About 39.6% of adolescent smokers were not denied buying cigarettes from commercial vendors due to an age restriction, particularly for older adolescents and those in the Eastern Mediterranean region (**Table 1**). The proportion of adolescent smokers who were not denied buying cigarettes by commercial vendors varied largely across countries (**Figure 2** and Table S3 in the **Online Supplementary Document**).

**Figure 3:**
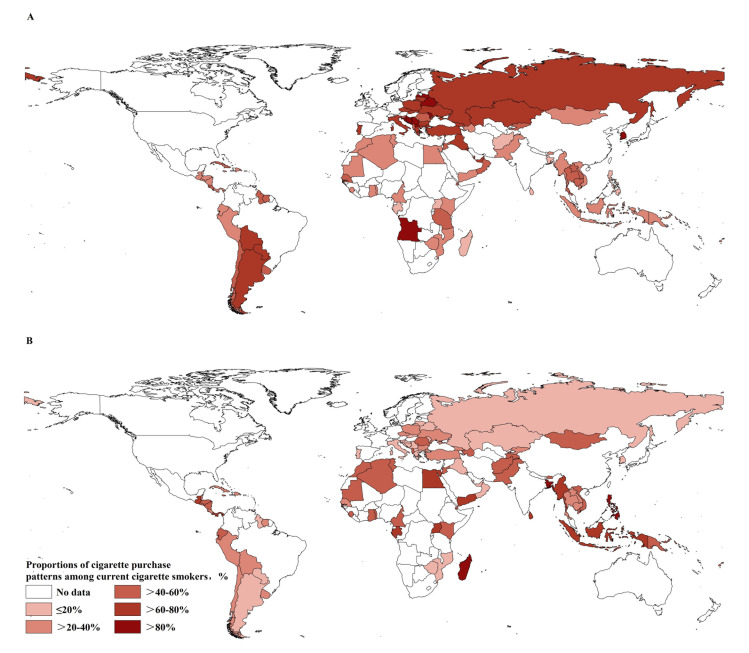
Proportions of adolescent smokers across countries who purchased cigarettes **Panel A:** in packs and **Panel B:** as single sticks across countries.

As shown in **Table 1****,** adolescent smokers usually bought cigarettes in packs (46.3%) or as individual sticks (37.1%). The proportion of adolescent smokers purchasing cigarettes per pack was highest in the European region, whereas the proportion of adolescents purchasing cigarettes as individual sticks was the highest in the South-East Asian region. The proportion of adolescent smokers purchasing cigarettes in packs was higher in upper-middle-income and high-income countries compared to lower-middle-income countries, with an inverse trend found for purchasing cigarettes as individual sticks. The proportion of adolescent smokers purchasing cigarettes in packs (**Figure 3**, Panel A, Table S4 in the **Online Supplementary Document**) and as individual sticks also varied largely across countries (**Figure 3**, Panel B, Table S4 in the **Online Supplementary Document**).

**Figure 4:**
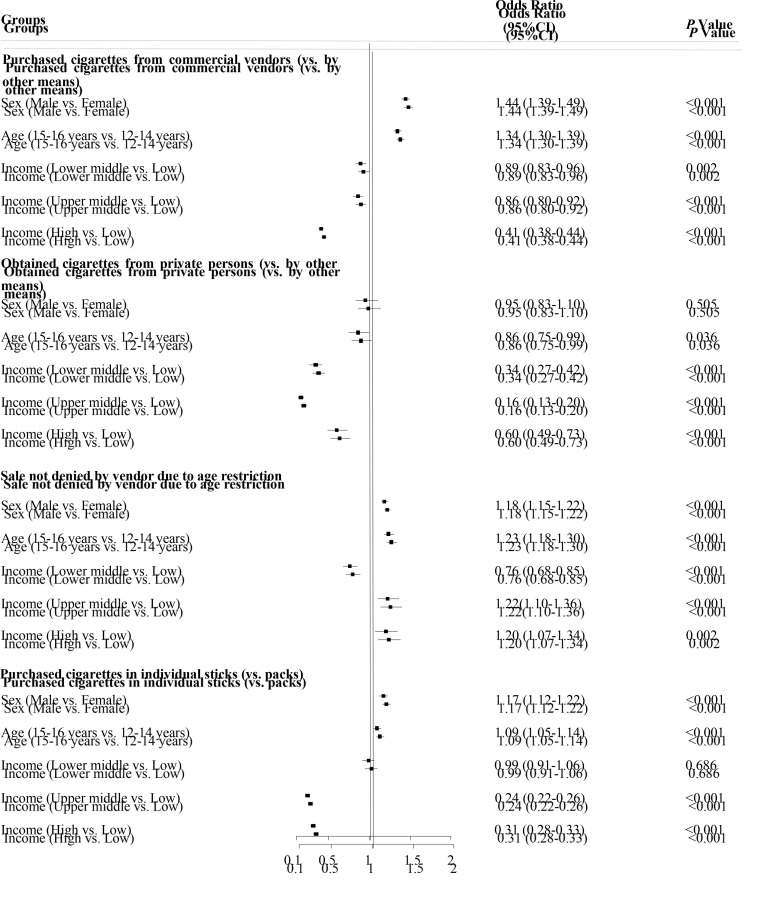
Multivariable analysis of potential factors associated with cigarette access and purchase patterns for adolescent smokers.

As shown in **Figure 4**, males (vs females, adjusted odds ratio (aOR) = 1.44, 95% CI = 1.39-1.49) and older adolescents (vs younger ones, aOR = 1.34, 95% CI = 1.30-1.39) were more likely to get cigarettes from commercial vendors, while younger adolescents (vs older ones, aOR = 1.16, 95% CI = 1.01-1.33) were more likely to get cigarettes from private persons. Adolescent smokers from low-income countries were more likely to get cigarettes from both commercial vendors (*P* < 0.01) and private persons (*P* < 0.01). Males, older adolescents, and adolescents from upper-middle-income or high-income countries were more likely to not be denied buying cigarettes due to age restrictions (all *P* < 0.01). Males, older adolescents, and those from low-income countries were more likely to purchase cigarettes as individual sticks (*P* < 0.001).

## DISCUSSION

In 49 856 adolescent cigarette smokers aged 12-16 years from 140 countries, over half obtained cigarettes from commercial vendors, and approximately one-third obtained cigarettes from private persons during the past 30 days. Males, older age, and persons with low income were associated with buying cigarettes from commercial vendors, while younger age persons and those of low income were associated with getting cigarettes from private persons. Two-fifths of adolescent smokers were not denied buying cigarettes from commercial vendors, and this occurred more among males, older adolescents, and those in upper-middle-income or high-income countries. Nearly half (46.3%) of adolescent smokers purchased cigarettes in packs and 37.1% as single sticks. Males, older adolescents, and those in low-income countries were more likely to buy cigarettes as single sticks.

Our findings update previous results from a study based on 45 countries from the GYTS between 2013 and 2014, which similarly showed that nearly half of the adolescent smokers aged 13-15 years got cigarettes from a store, street vendor, or kiosk [13], suggesting that reducing cigarette access from commercial vendors (ie, the supply side) is the core mission for the tobacco control among adolescent smokers. We also found that 39.6% of adolescent smokers were not denied buying cigarettes from commercial vendors, implying that age check by retailers was not fully applied, especially among males, older adolescents, and those in upper-middle-income or high-income countries. The WHO Framework Convention on Tobacco Control (FCTC) Article 16 recommends that cigarettes are not sold to minors and that they should not be sold through vending machines. However, adolescents can alternatively get cigarettes from other sources [15]. We indeed found that about a third of adolescent smokers got cigarettes from private persons (possibly those aged 18-20 years [22]). It was, however, shown that, when the age checking by retailers is not well enforced, adolescents purchase cigarettes more often from retail shops [15].

In this study, 46.3% of adolescent smokers purchased cigarettes in packs, especially in the European region (68.5%) and in high-income countries (58.7%), while 37.1% purchased cigarettes as individual sticks, and the proportion buying cigarettes as individual sticks was higher in regions of South-East Asia (57.2%) and Africa (48.5%), and in low-income (45.4%) and lower-middle-income countries (48.0%) compared to other countries. A previous study based on GYTS conducted between 2013 and 2014 in 45 countries similarly showed that nearly 40% of adolescent smokers purchased individual cigarettes in most countries in regions of South-East Asia and Africa, and approximately 50% of adolescent smokers purchased cigarettes in packs in most countries in the European region [13]. These findings suggest that adolescents in low- and middle-income countries, who are more sensitive to cigarettes price, are more inclined to buy cigarettes as single sticks, but national laws also tend to ban sales as individual sticks in low- or lower-middle vs high-income countries.

Overall, our findings suggest that tobacco control measures recommended by the WHO FCTC [23] should be strengthened. Regarding cigarette purchases by minors, supply-side interventions are particularly relevant. This includes harshly tackling illicit tobacco trade, a ban on tobacco sales to minors, raising the legal age to sell cigarettes to persons up to the age of 21 years [24], mandatory age checking by cigarette retailers, sales of tobacco products authorized only for retailers licensed to sell tobacco products, a total ban of cigarette vending machines, a ban on selling cigarette by units or in small packets, measures to control the age of cigarette buyers on the Internet or in special events (eg, when cigarettes are distributed for free at some events), and mandatory measures to avoid that cigarette packets are directly accessible by the clients in stores (eg, cigarette placed behind the counter). Interventions that reduce the demand for tobacco products also need to be reinforced, such as a high tax on tobacco products (eg, at least 70% of the retail price of cigarettes), a ban on tobacco advertising, promotion, and sponsorship, and a ban on smoking in enclosed and other places. Beyond the obviously central role of prohibiting sales of tobacco products to minors, a ban on sales by units (vs full packets) [25,26], and high tax on tobacco products can be particularly effective among adolescents, as they are highly sensitive to cigarette cost [27]. However, strict enforcement of laws prohibiting sales of tobacco products to minors can also lead adolescents to purchase cigarettes from private vendors, which is difficult to regulate and control. Other factors can also have an important role in tobacco use by adolescents, such as large social tolerance to tobacco use in some countries [28] and interference by the tobacco industry in the process to establish stringent tobacco control policies. This emphasizes the need for continued health education programs to increase awareness of the hazards of tobacco use targeting both youth and adults.

We found that the main sources of cigarette access were commercial vendors and private persons across countries, which may guide the development and enforcement of public policies and programmes on prevention and control of cigarette access, according to national circumstances. The proportion differences in sex, age, WHO region, and income level may also contribute to more specific and efficient strategies. When the bans and regulations are strongly enforced, adolescents possibly smoke less as a result. Previous studies have confirmed the adverse effect of cigarette use on health outcomes, such as cardiovascular diseases [17]. We speculate that national policies and programmes on limiting cigarette access among adolescents according to our findings may have a potentially important role in originally reducing the short-term and long-term disease burdens.

This study has several strengths. First, we used the most recent available data from 140 nationally representative surveys using a standardized methodology, which makes the results broadly generalizable and comparable across countries. Second, we quantified access to cigarettes and purchase patterns among adolescent smokers by age, sex, country, region, and national income level. However, this study also has several limitations. First, data came from a self-reported questionnaire and may be subject to over- or under-reporting. However, the use of the GYTS questions has been shown to have good reliability for different domains [20,21]. Second, only access to cigarettes (vs other tobacco products) was examined in GYTS questionnaires. Third, access to cigarettes through private persons and vending machines was not examined in detail [16]. Fourth, adolescents aged 12-16 years only were included in this study, which may influence the generalization of our findings to other age groups. Fifth, this study included only 140 countries due to the unavailability of data on cigarette access from other countries. Sixth, data from one or more cities were used in six of 140 countries, which could not represent the proportion of the whole country. Seventh, the proportions of cigarette access and purchase patterns varied largely across countries. However, the proportions according to WHO regions were pooled to allow direct comparisons between our findings and those in the previous studies [13,15]. Eighth, GYTS is a school-based survey, and the use of samples of students in schools might bias the true proportion of the whole adolescents because students out of school are more likely to have undesirable behaviour than those in schools [29]. Ninth, survey years varied across countries between 2010 and 2018, which may limit the direct comparisons. However, the survey years in 122 of 140 countries (87.5%) were distributed between 2013 and 2018. Tenth, the questions on the sources of cigarette purchases and purchase patterns referred to the last time ones bought cigarettes during the past 30 days, which may not reflect the usual way that adolescents bought cigarettes.

## CONCLUSIONS

This study assessed various channels through which adolescent smokers accessed cigarettes in many countries. Our findings highlight the need to strengthen measures to limit the access to cigarettes among adolescents, including enforcement of age checking by cigarettes retailers, ensuring that cigarettes are sold only through licensed vendors, and banning the sale of cigarettes by units, as advised by the WHO Framework Convention on Tobacco Control.

## Additional material


Online Supplementary Document
