# Supplementary material for: Cigarette access and purchase patterns among adolescent smokers aged 12-16 years in 140 countries/territories, Global Youth Tobacco Survey 2010-2018
Source: J Glob Health. 2022 Dec 21;12:04101. doi: 10.7189/jogh.12.04101 (PMC9767304; doi:10.7189/jogh.12.04101)
Supplement: Online Supplementary Document [file jogh-12-04101-s001.pdf]

ONLINE SUPPLEMENTARY DOCUMENT

Cigarette access and purchase patterns among adolescent smokers aged 12-16 years in 140 countries/territories, Global Youth Tobacco Survey 2010-2018

Authors

Jiahong Sun, *PhD*;[1] Bo Xi, *PhD*;[1*] Chuanwei Ma, *PhD candidate*;[1] Zilin Li, *MS candidate*; [1] Min Zhao, *PhD*;[2] Pascal Bovet, *MD* [3]

Affiliations

1 Department of Epidemiology, School of Public Health, Qilu Hospital, Cheeloo College of Medicine, Shandong University, Jinan, Shandong, China

2 Department of Nutrition and Food Hygiene, School of Public Health, Cheeloo College of Medicine, Shandong University, Jinan, Shandong, China

3 Center for Primary Care and Public Health (Unisanté), University of Lausanne, Lausanne, Switzerland

[*] **Correspondence to:** Dr. Bo Xi, Department of Epidemiology, School of Public Health, Cheeloo College of Medicine, Shandong University, 44 Wen Hua Xi Road, Jinan, 250012, China. Tel/Fax: +86-531-88382141; Email: xibo2007@126.com

Table S1. Statistical power to detect significant difference between groups

	Source of cigarette purchase				No refusal of sale from commercial vendors due to age	Purchase patterns		
	Bought in stores/shops/street vendors/kiosks	Got from private persons	Bought from vending machine	Other ways		Pack	Individual sticks	Other patterns
Sex	1.000	1.000	0.225	1.000	1.000	1.000	1.000	1.000
Age group	1.000	1.000	1.000	1.000	1.000	1.000	0.349	1.000
WHO region (America† as ref.)								
Africa	1.000	1.000	1.000	0.198	0.312	1.000	1.000	1.000
Eastern Mediterranean	1.000	1.000	1.000	0.893	1.000	0.999	0.990	0.902
Europe	1.000	1.000	1.000	0.413	1.000	1.000	1.000	0.996
South-East Asia	1.000	1.000	0.067	1.000	0.054	1.000	1.000	0.057
Western Pacific	0.645	1.000	0.482	0.448	1.000	0.999	0.219	0.989
World Bank income (Lower middle† as ref.)								
Low	0.999	0.084	0.987	1.000	0.473	0.812	0.714	1.000
Upper middle	1.000	1.000	0.385	0.894	1.000	1.000	1.000	0.999
High	1.000	1.000	1.000	1.000	0.978	1.000	1.000	0.999

†America (with an intermediate proportion) and lower middle (with an intermediate income) were as references.

Table S2. Characteristics of the Global Youth Tobacco Surveys of adolescents aged 12-16 years in 140 countries/territories (data from 2010-2018)

Country/territory	Survey year	Sample size of population	Prevalence of current cigarette smoking (weighted), %	Number of current cigarette smokers	Proportion of boys among current cigarette users, %	Age, years
**Africa**						
Algeria	2013	5005	5.7	343	93.7	12-16
Angola	2010	1054	0.5	9	100.0	12-16
Cameroon	2014	2215	3.2	128	80.4	12-16
Comoros	2015	2007	4.2	139	77.3	12-16
Gabon	2014	1134	5.2	67	63.2	12-16
Gambia	2017	8784	4.4	580	78.3	12-16
Ghana	2017	4994	1.7	141	50.7	12-16
Kenya	2013	1662	4.0	77	71.4	12-16
Madagascar	2018	1933	7.8	251	79.3	12-16
Mauritania	2018	2872	8.3	349	57.0	12-16
Mauritius	2016	3711	12.4	491	76.1	12-16
Mozambique	2013	3846	1.2	73	44.6	12-16
Sao Tome and Principe	2010	4698	3.3	186	72.5	12-16
Senegal	2013	1138	2.6	52	75.2	12-16
Seychelles	2015	2158	12.6	327	60.2	12-16
Sierra Leone	2017	4260	2.4	138	81.0	12-16
South Africa	2011	5721	13.4	809	53.9	12-16
Togo	2013	3978	2.4	219	85.7	12-16
Uganda	2018	2828	2.8	117	62.6	12-16
United Republic of Tanzania	2016	3339	0.8	36	65.5	12-16
Zambia	2011	2162	4.8	133	47.6	12-16

Zimbabwe	2014	5195	10.8	402	60.3	12-16
**America**						
Antigua and Barbuda	2017	1912	1.0	25	58.5	12-16
Argentina	2018	1324	17.9	248	43.2	12-16
Bahamas	2013	1204	2.6	46	65.6	12-16
Barbados	2013	1582	4.6	106	60.7	12-16
Belize	2014	1640	6.5	115	63.4	12-16
Bolivia	2018	4077	5.8	341	59.2	12-16
Brazil	2011	3185	6.2	225	50.1	12-16
Chile	2016	10223	16.7	1622	35.4	12-16
Costa Rica	2013	2723	4.8	144	59.3	12-16
Cuba	2018	3836	8.2	387	59.9	12-16
Dominican Republic	2016	1143	0.7	32	56.6	12-16
Ecuador	2016	4475	1.8	449	67.2	12-16
El Salvador	2015	2774	8.8	300	58.8	12-16
Grenada	2016	1906	4.6	114	56.6	12-16
Guatemala	2015	3545	10.2	466	59.0	12-16
Guyana	2015	1390	4.4	93	80.0	12-16
Honduras	2016	3117	3.5	187	53.6	12-16
Jamaica	2017	1259	9.5	156	42.5	12-16
Mexico	2011	2259	13.6	363	54.8	12-16
Nicaragua	2014	3626	10.1	466	53.0	12-16
Panama	2017	2449	3.0	94	55.8	12-16
Paraguay	2014	6172	2.5	219	51.5	12-16
Peru	2014	3234	6.7	332	60.0	12-16
Saint Kitts and Nevis	2010	658	4.0	30	60.5	12-16
Saint Lucia	2017	1397	4.7	80	60.5	12-16
Saint Vincent and the Grenadines	2018	1215	3.4	57	59.8	12-16

Suriname	2016	1661	6.6	143	66.4	12-16
Trinidad and Tobago	2017	3345	5.4	198	67.3	12-16
Uruguay	2014	4421	6.4	299	38.9	12-16
Venezuela	2010	2156	4.2	93	55.4	12-16
**Eastern Mediterranean**						
Afghanistan	2017	1352	2.4	39	93.8	12-16
Bahrain	2015	3097	8.2	228	76.6	12-16
Djibouti	2013	1411	5.2	100	77.3	12-16
Egypt	2014	2065	3.7	116	88.1	12-16
Gaza Strip	2013	1890	5.5	116	66.9	12-16
Iraq	2014	1459	5.3	114	79.9	12-16
Jordan	2014	1935	10.4	229	74.8	12-16
Kuwait	2016	2113	7.6	193	88.1	12-16
Lebanon	2011	2008	12.2	243	73.4	12-16
Libyan Arab Jamahiriya	2010	1678	3.5	73	73.1	12-16
Morocco	2016	3576	1.4	76	84.2	12-16
Oman	2016	1948	1.6	43	74.0	12-16
Pakistan	2013	7183	1.4	178	85.5	12-16
Qatar	2018	1887	4.7	129	75.1	12-16
Saudi Arabia	2010	2132	8.7	193	70.1	12-16
Syrian Arab Republic	2010	1495	6.8	105	74.0	12-16
Tunisia	2017	2302	6.4	166	91.1	12-16
United Arab Emirates	2013	3878	6.1	250	80.5	12-16
UNRWA GAZA	2013	1543	8.5	167	65.1	12-16
UNRWA Jordan	2014	1209	11.2	143	76.1	12-16
UNRWA Lebanon	2013	1328	9.9	148	74.3	12-16
UNRWA west bank	2014	1214	17.6	254	62.5	12-16
West BANK	2016	1329	15.8	255	82.2	12-16
Yemen	2014	1739	4.5	98	82.7	12-16

**Europe**						
Albania	2015	4258	6.1	338	74.8	12-16
Azerbaijan	2016	2087	3.1	79	90.2	12-16
Belarus	2015	2831	6.6	209	46.5	12-16
Bosnia and Herzegobina	2013	10963	11.3	967	60.9	12-16
Bulgaria	2015	3754	20.7	820	43.1	12-16
Croatia (Hrvatska)	2016	3055	14.9	542	50.3	12-16
Cyprus	2011	983	13.6	143	72.4	12-16
Czech Republic	2016	3696	13.6	543	46.5	12-16
Finland	2012	4616	15.3	763	50.4	12-16
Georgia	2017	1204	6.0	95	74.3	12-16
Greece	2013	4294	9.1	458	51.7	12-16
Italy	2018	1593	20.0	347	43.5	12-16
Kazakhstan	2014	1937	1.3	36	60.4	12-16
Kosovo	2016	4724	2.3	207	74.6	12-16
Kyrgyzstan	2014	3985	1.6	145	81.6	12-16
Latvia	2014	4039	15.6	657	49.6	12-16
Lithuania	2018	2750	15.5	535	50.6	12-16
Macedonia	2016	4753	6.3	374	59.4	12-16
Malta	2017	1197	5.2	67	48.4	12-16
Montenegro	2018	3960	4.7	259	53.3	12-16
Poland	2016	4579	17.6	833	49.5	12-16
Portugal	2013	9122	8.7	966	49.1	12-16
Republic of Moldova	2013	3575	5.8	269	80.3	12-16
Romania	2017	5061	6.3	394	57.4	12-16
Russian Federation	2015	5994	7.4	591	49.9	12-16
San Marino	2018	595	6.9	43	57.8	12-16
Serbia	2017	3624	12.8	494	46.3	12-16
Slovakia	2016	3723	15.5	606	44.7	12-16

Slovenia	2017	2388	7.6	204	41.8	12-16
Tajikistan	2014	2988	0.6	26	59.0	12-16
Turkey	2017	106466	10.4	12854	61.6	12-16
Ukraine	2017	3690	6.7	252	57.3	12-16
**South-East Asia**						
Bangladesh	2013	3111	2.0	51	99.9	12-16
Bhutan	2013	1855	12.6	263	71.5	12-16
Indonesia	2014	5414	14.6	846	94.4	12-16
Maldives	2011	2049	5.2	120	38.3	12-16
Myanmar	2016	3292	7.8	357	92.4	12-16
Nepal	2011	2247	4.0	95	81.0	12-16
Sri Lanka	2015	1472	1.3	23	100.0	12-16
Thailand	2015	1767	8.4	189	74.7	12-16
Timor-Leste	2013	1347	23.4	464	80.9	12-16
**Western Pacific**						
Brunei Darussalam	2013	1379	9.4	126	81.9	12-16
Cambodia	2016	2706	0.6	18	80.1	12-16
Cook Islands	2016	433	15.2	82	66.7	12-16
Fiji	2016	2332	7.4	193	63.2	12-16
Guam	2017	1664	8.4	161	58.3	12-16
Kiribati	2018	1690	19.9	337	66.2	12-16
Laos	2016	5304	5.6	377	84.9	12-16
Macao (China)	2015	1620	2.7	47	55.8	12-16
Marshall Islands	2016	1859	12.9	288	70.6	12-16
Micronesia	2013	3115	24.7	899	61.5	12-16
Mongolia	2014	6633	3.8	383	71.8	12-16
New Caledonia	2010	845	30.5	222	45.5	12-16
Northern Mariana Islands	2014	2012	9.4	212	61.5	12-16
Palau	2017	959	29.0	316	53.2	12-16

Papua New Guinea	2016	1590	20.8	417	65.5	12-16
Philippines	2015	7181	10.8	885	69.0	12-16
Samoa	2017	1373	9.3	134	82.5	12-16
South Korea	2013	4098	4.3	177	75.9	12-16
Tokelau	2014	82	47.2	43	50.6	12-16
Tonga	2010	1765	26.5	449	56.5	12-16
Tuvalu	2018	551	11.9	89	80.6	12-16
Vanuatu	2017	1484	13.8	243	62.1	12-16
Viet Nam	2014	3455	2.1	81	95.8	12-16
**Total**	—	509541	7.7	49856	65.6	12-16

UNRWA, United Nations Relief and Works Agency.

Table S3. Proportions of methods of obtaining cigarettes (during the past 30 days) among current cigarette smokers by country, 2010-2018

Country/territory	Survey year	Bought in store/shop/ street vendor/kiosk†	Got from someone else†	Bought from vending machine†	Other way†	No refusal of sales from commercial vendors due to age†
**Africa**						
Algeria	2013	60.2(52.5-67.4)	32.2(24.8-40.7)	—	7.6(5.0-11.5)	57.7(51.3-63.9)
Angola	2010	24.4(4.8-67.3)	38.3(8.4-80.7)	—	37.3(6.2-84.4)	13.9(2.0-56.8)
Cameroon	2014	57.8(48.3-66.7)	18.7(12.2-27.6)	4.2(1.5-11.3)	19.3(12.8-28.1)	38.3(29.7-47.7)
Comoros	2015	60.2(46.2-72.7)	30.1(20.3-42.1)	—	9.8(5.4-17.0)	40.5(33.8-47.5)
Gabon	2014	68.6(51.8-81.7)	31.4(18.3-48.2)	—	—	45.7(30.3-61.9)
Gambia	2017	62.6(57.1-67.9)	1.8(0.7-4.7)	11.9(8.1-17.2)	23.6(18.9-29.0)	35.5(31.2-40.1)
Ghana	2017	70.6(51.8-84.3)	15.5(7.2-30.3)	—	13.9(5.9-29.2)	32.7(21.6-46.1)
Kenya	2013	49.6(39.7-59.6)	37.6(27.6-48.8)	—	12.8(6.9-22.5)	54.3(37.6-70.0)
Madagascar	2018	54.6(41.3-67.3)	30.3(19.0-44.6)	—	15.0(6.6-30.6)	53.4(39.7-66.6)
Mauritania	2018	74.8(58.5-86.2)	11.8(7.3-18.7)	—	13.4(4.4-34.4)	37.2(29.2-45.9)
Mauritius	2016	52.1(42.4-61.7)	37.8(28.9-47.5)	—	10.1(6.6-15.2)	42.6(37.2-48.1)
Mozambique	2013	61.2(47.0-73.8)	19.6(10.4-33.9)	—	19.2(9.8-33.9)	29.3(18.9-42.3)
Sao Tome and Principe	2010	11.8(6.7-16.9)	52.9(45.0-60.8)	5.9(2.2-9.6)	29.4(22.2-36.6)	29.4(23.7-35.1)
Senegal	2013	39.4(21.2-61.2)	56.8(34.4-76.7)	—	3.8(0.9-14.4)	29.7(15.0-50.5)
Seychelles	2015	50.0(43.8-56.1)	36.3(30.1-43.0)	—	13.7(9.7-19.1)	37.6(31.9-43.7)
Sierra Leone	2017	66.7(50.7-79.6)	25.2(14.0-41.1)	—	8.1(3.6-16.9)	47.2(31.3-63.6)
South Africa	2011	55.0(49.3-60.5)	26.8(23.1-30.9)	2.2(1.2-4.0)	16.1(12.4-20.5)	47.2(43.3-51.1)
Togo	2013	57.8(41.8-72.3)	23.8(14.8-36.1)	—	18.4(10.9-29.3)	22.0(15.3-30.5)
Uganda	2018	31.4(16.8-50.9)	36.5(22.5-53.2)	—	32.2(17.8-50.8)	22.1(12.3-36.6)
United Republic of Tanzania	2016	91.1(68.4-98.0)	—	—	8.9(2.0-31.6)	42.6(27.2-59.5)
Zambia	2011	22.3(19.9-25.0)	51.5(46.6-56.2)	12.6(9.8-16.2)	13.6(12.7-14.5)	30.6(30.5-30.8)

Zimbabwe	2014	55.4(39.9-70.0)	7.5(4.0-13.7)	14.4(5.4-32.8)	22.7(10.3-42.9)	29.5(20.9-39.9)
**America**						
Antigua and Barbuda	2017	38.3(19.4-61.5)	43.4(24.5-64.5)	5.1(0.7-28.9)	13.3(4.3-34.2)	40.5(22.0-62.2)
Argentina	2018	70.4(49.8-85.0)	25.1(12.5-43.8)	0.8(0.2-3.3)	3.8(1.7-8.2)	63.9(51.3-74.8)
Bahamas	2013	44.3(29.0-60.7)	36.7(22.7-53.4)	5.7(1.3-21.2)	13.3(5.4-29.4)	48.7(34.6-63.1)
Barbados	2013	23.7(13.7-37.9)	57.3(44.4-69.3)	1.1(0.2-7.5)	17.9(10.7-28.4)	30.2(21.8-40.2)
Belize	2014	44.2(35.2-53.6)	46.4(38.9-54.1)	—	9.4(5.5-15.5)	49.8(42.3-57.4)
Bolivia	2018	61.9(54.5-68.8)	22.1(16.3-29.3)	2.2(1.0-4.8)	13.8(9.2-20.1)	36.3(31.3-41.6)
Brazil	2011	29.9(23.8-36.8)	49.6(41.1-58.2)	—	20.5(14.4-28.3)	31.8(24.5-40.2)
Chile	2016	44.5(40.8-48.2)	42.8(39.2-46.5)	—	12.7(10.5-15.2)	39.2(35.6-42.9)
Costa Rica	2013	23.8(16.7-32.8)	66.9(57.3-75.3)	—	9.3(5.5-15.3)	29.9(23.0-37.7)
Cuba	2018	67.1(57.2-75.7)	12.7(7.5-20.9)	1.9(0.9-4.4)	18.2(12.3-26.1)	43.8(36.0-51.9)
Dominican Republic	2016	54.9(21.1-84.7)	14.0(1.7-60.5)	12.2(1.5-56.6)	19.0(4.6-53.1)	32.3(15.1-56.2)
Ecuador	2016	48.8(33.4-64.5)	34.2(23.8-46.5)	2.8(0.6-11.3)	14.1(7.6-24.8)	28.9(25.1-33.1)
El Salvador	2015	43.5(37.3-49.9)	40.9(34.4-47.6)	1.0(0.3-3.7)	14.6(10.3-20.4)	43.1(37.8-48.6)
Grenada	2016	31.0(22.2-41.4)	44.8(34.3-55.7)	—	24.2(14.0-38.5)	42.7(32.6-53.4)
Guatemala	2015	83.6(79.6-87.0)	11.3(8.4-15.0)	—	5.1(3.3-7.8)	51.0(44.5-57.5)
Guyana	2015	55.0(34.7-73.7)	26.6(12.6-47.5)	2.6(0.3-17.1)	15.9(10.0-24.3)	25.4(18.0-34.5)
Honduras	2016	60.4(51.6-68.5)	25.8(16.7-37.6)	0.7(0.2-2.9)	13.1(7.3-22.4)	28.4(20.8-37.5)
Jamaica	2017	55.8(46.9-64.7)	24.2(16.5-31.9)	3.3(0.1-6.5)	16.7(10.0-23.4)	44.0(36.1-51.9)
Mexico	2011	42.6(36.3-49.1)	53.4(46.8-59.8)	1.2(0.5-2.6)	2.8(1.4-5.6)	37.4(30.7-44.6)
Nicaragua	2014	57.9(51.0-64.5)	36.9(30.3-44.0)	1.4(0.4-4.6)	3.8(2.2-6.8)	38.2(32.4-44.3)
Panama	2017	37.0(26.4-49.0)	40.7(31.4-50.6)	3.0(0.7-11.7)	19.4(10.9-32.2)	31.3(23.8-39.9)
Paraguay	2014	62.2(49.2-73.7)	0.1(0.0-0.9)	—	37.7(26.2-50.7)	50.9(43.9-57.8)
Peru	2014	65.9(50.7-78.4)	24.1(14.8-36.7)	1.0(0.3-4.1)	8.9(5.0-15.4)	25.8(18.2-35.2)
Saint Kitts and Nevis	2010	9.7(2.9-28.0)	66.0(43.7-82.9)	—	24.3(9.4-49.7)	26.1(12.7-46.1)
Saint Lucia	2017	37.1(25.7-50.2)	41.8(31.1-53.4)	—	21.1(12.6-33.1)	33.0(22.3-45.6)
Saint Vincent and the Grenadines	2018	31.2(16.6-50.8)	56.9(39.9-72.4)	3.8(0.6-21.8)	8.0(2.5-23.2)	25.1(14.8-39.3)

Suriname	2016	44.3(35.9-53.0)	36.9(29.0-45.4)	—	18.9(12.7-27.1)	42.6(35.8-49.8)
Trinidad and Tobago	2017	47.3(38.4-56.3)	31.5(22.2-42.5)	2.4(0.6-9.2)	18.8(13.6-25.5)	42.3(35.6-49.4)
Uruguay	2014	65.7(58.7-72.1)	32.1(25.2-39.8)	—	2.2(0.9-5.1)	54.6(49.8-59.3)
Venezuela	2010	26.4(19.0-35.5)	40.4(30.5-51.1)	1.4(0.2-9.3)	31.8(24.9-39.6)	—
**Eastern Mediterranean**						
Afghanistan	2017	51.6(34.2-68.7)	14.1(4.9-34.1)	—	34.3(19.9-52.4)	42.2(26.3-60.0)
Bahrain	2015	64.9(54.5-74.1)	22.0(14.7-31.4)	—	13.1(8.2-20.4)	44.6(37.2-52.2)
Djibouti	2013	73.6(57.1-85.3)	15.5(8.1-27.4)	—	11.0(5.3-21.4)	43.1(31.1-56.0)
Egypt	2014	82.7(63.2-93.0)	7.6(4.2-13.4)	2.8(0.4-18.2)	6.8(1.7-23.9)	61.6(41.4-78.5)
Gaza Strip	2013	46.4(35.6-57.5)	38.4(30.3-47.3)	—	15.2(8.1-26.6)	31.8(15.7-53.8)
Iraq	2014	56.6(44.9-67.7)	37.2(28.2-47.1)	—	6.2(2.7-13.7)	32.2(21.1-45.7)
Jordan	2014	51.9(43.5-60.2)	21.3(14.7-29.7)	26.2(21.5-31.5)	0.6(0.1-3.7)	49.4(35.7-63.2)
Kuwait	2016	88.4(81.8-92.8)	3.7(1.8-7.5)	—	7.8(4.2-14.0)	60.5(53.3-67.2)
Lebanon	2011	34.0(26.8-42.1)	35.0(29.4-41.0)	—	31.0(23.8-39.2)	55.6(47.9-63.0)
Libyan Arab Jamahiriya	2010	32.6(18.7-50.5)	35.9(23.5-50.7)	—	31.4(19.3-46.7)	42.5(28.5-57.7)
Morocco	2016	69.6(48.4-84.9)	23.3(9.9-45.6)	—	7.1(2.0-22.6)	45.8(32.4-59.8)
Oman	2016	51.0(33.1-68.6)	16.0(8.7-27.6)	—	33.0(22.4-45.7)	37.1(24.1-52.4)
Pakistan	2013	70.2(52.2-83.6)	—	—	29.8(16.4-47.8)	22.4(10.9-40.4)
Qatar	2018	56.9(49.2-64.4)	25.8(17.3-36.6)	—	17.3(10.9-26.4)	38.7(32.2-45.6)
Saudi Arabia	2010	25.6(17.4-36.0)	55.1(45.6-64.3)	—	19.3(13.6-26.5)	57.0(48.3-65.3)
Syrian Arab Republic	2010	36.7(24.2-51.2)	39.3(28.5-51.4)	—	24.0(15.2-35.7)	48.1(35.9-60.5)
Tunisia	2017	60.7(53.1-67.9)	30.7(23.4-39.2)	—	8.5(4.7-15.0)	55.0(47.4-62.4)
United Arab Emirates	2013	52.2(43.5-60.7)	28.1(21.7-35.4)	—	19.8(15.6-24.8)	40.9(34.2-47.9)
UNRWA GAZA	2013	40.8(28.6-54.2)	36.1(24.2-50.0)	3.9(2.7-5.5)	19.3(13.8-26.3)	37.2(28.9-46.3)
UNRWA Jordan	2014	58.0(45.6-69.5)	23.9(16.9-32.6)	1.1(0.2-6.4)	17.0(9.9-27.4)	52.3(44.0-60.5)
UNRWA Lebanon	2013	52.7(41.9-63.3)	34.4(23.3-47.5)	—	12.9(7.9-20.3)	51.1(40.5-61.6)
UNRWA west bank	2014	43.0(29.5-57.7)	36.4(26.8-47.2)	2.4(0.9-5.9)	18.2(13.0-24.8)	45.2(36.6-54.0)
West BANK	2016	55.5(44.4-66.1)	30.8(22.4-40.7)	—	13.7(9.7-19.0)	53.4(43.3-63.2)
Yemen	2014	44.0(26.0-63.9)	48.8(27.1-71.0)	—	7.2(2.4-19.8)	37.9(20.5-59.1)

**Europe**						
Albania	2015	75.7(71.9-79.2)	17.3(13.8-21.6)	—	7.0(4.3-11.2)	52.2(46.4-57.8)
Azerbaijan	2016	89.3(74.0-96.1)	4.9(1.7-13.3)	—	5.8(0.9-28.8)	58.5(49.6-66.7)
Belarus	2015	34.7(29.1-40.8)	41.0(32.5-50.0)	—	24.3(18.3-31.6)	24.0(16.9-32.8)
Bosnia and Herzegobina	2013	61.0(56.4-65.5)	32.1(28.3-36.0)	—	6.9(4.8-9.8)	64.2(60.0-68.1)
Bulgaria	2015	72.0(67.1-76.4)	24.4(19.7-29.7)	—	3.7(2.0-6.5)	54.9(49.6-60.1)
Croatia (Hrvatska)	2016	64.9(56.9-72.1)	24.4(19.1-30.6)	0.3(0.0-2.2)	10.4(6.2-16.9)	39.2(35.5-43.0)
Cyprus	2011	49.3(48.5-50.0)	29.9(29.7-30.0)	—	20.9(20.0-21.8)	72.1(66.1-77.3)
Czech Republic	2016	50.9(44.3-57.6)	32.0(26.4-38.1)	2.1(1.0-4.3)	15.0(12.0-18.6)	40.6(34.6-46.8)
Finland	2012	5.6(4.0-7.8)	46.9(42.7-51.2)	—	47.4(42.7-52.3)	8.9(7.0-11.3)
Georgia	2017	74.5(60.3-84.9)	12.0(6.5-21.1)	1.3(0.2-8.8)	12.2(6.1-23.0)	32.4(23.9-42.3)
Greece	2013	69.1(63.7-74.0)	23.8(19.4-28.8)	1.3(0.4-4.0)	5.9(3.9-8.7)	63.2(56.5-69.5)
Italy	2018	23.5(17.8-30.3)	61.3(54.9-67.4)	15.2(9.7-23.0)	—	36.3(30.4-42.5)
Kazakhstan	2014	50.3(28.9-71.6)	27.0(16.1-41.8)	—	22.6(10.3-42.7)	41.9(24.7-61.4)
Kosovo	2016	75.6(66.5-82.9)	14.0(8.2-22.7)	—	10.4(5.6-18.5)	41.1(30.3-52.8)
Kyrgyzstan	2014	82.3(73.1-88.9)	14.9(8.8-24.0)	—	2.8(0.8-9.3)	34.1(24.0-46.0)
Latvia	2014	26.6(21.3-32.8)	49.4(43.7-55.0)	—	24.0(20.1-28.4)	33.0(29.3-37.0)
Lithuania	2018	19.9(15.4-25.3)	53.2(48.1-58.1)	3.5(1.8-6.7)	23.4(18.4-29.3)	29.8(25.2-34.8)
Macedonia	2016	74.5(67.9-80.2)	22.6(17.3-28.9)	1.2(0.4-3.4)	1.7(0.7-4.0)	49.9(43.8-56.1)
Malta	2017	27.4(16.3-38.5)	30.6(19.1-42.1)	21.0(10.9-31.1)	21.0(10.9-31.1)	50.7(38.9-62.5)
Montenegro	2018	64.0(57.9-69.7)	—	—	36.0(30.3-42.1)	51.6(44.4-58.7)
Poland	2016	37.4(33.0-42.0)	38.4(34.3-42.5)	—	24.3(20.8-28.1)	33.6(28.8-38.8)
Portugal	2013	25.6(22.2-29.3)	49.0(45.0-53.0)	17.8(14.7-21.3)	7.6(6.4-9.1)	41.7(37.5-46.0)
Republic of Moldova	2013	63.7(54.9-71.7)	27.5(19.5-37.4)	—	8.8(5.8-12.9)	28.6(23.5-34.4)
Romania	2017	71.3(66.4-75.7)	26.0(21.7-30.9)	—	2.7(1.5-4.8)	56.1(50.4-61.6)
Russian Federation	2015	41.3(33.0-50.1)	37.9(30.1-46.5)	2.5(0.8-6.9)	18.3(10.3-30.5)	25.4(18.9-33.3)
San Marino	2018	49.6(34.5-64.7)	14.6(5.1-34.9)	5.0(1.5-15.6)	30.8(19.6-45.0)	48.1(35.0-61.4)
Serbia	2017	72.0(67.9(76.1)	24.6(20.7-28.5)	—	3.4(1.8-5.0)	63.6(59.4-67.8)
Slovakia	2016	41.1(35.3-47.2)	47.9(41.1-54.7)	—	11.0(8.1-14.8)	40.2(35.2-45.4)

Slovenia	2017	47.1(35.5-58.9)	38.9(30.3-48.3)	—	14.0(8.7-21.7)	30.4(20.0-43.2)
Tajikistan	2014	77.6(62.5-87.8)	5.3(0.6-33.9)	17.1(7.9-32.9)	—	39.2(21.7-60.0)
Turkey	2017	65.6(63.6-67.5)	29.5(27.7-31.3)	—	4.9(4.3-5.6)	60.4(58.8-61.9)
Ukraine	2017	54.1(41.0-66.7)	19.1(11.6-29.9)	2.6(0.6-10.4)	24.2(18.2-31.4)	27.4(19.4-37.3)
**South-East Asia**						
Bangladesh	2013	85.5(59.8-95.9)	13.7(3.7-39.6)	—	0.7(0.2-3.2)	64.2(32.8-86.9)
Bhutan	2013	61.8(56.3-67.1)	30.4(24.9-36.4)	—	7.8(4.9-12.3)	33.7(27.6-40.4)
Indonesia	2014	63.7(56.8-70.2)	32.1(26.1-38.8)	0.4(0.1-1.3)	3.8(2.0-6.9)	43.7(39.2-48.3)
Maldives	2011	43.5(33.2-54.4)	33.7(25.0-43.6)	—	22.8(15.4-32.5)	54.2(46.0-62.2)
Myanmar	2016	57.9(51.3-64.1)	36.1(30.5-42.3)	—	6.0(3.5-9.9)	38.8(31.1-47.1)
Nepal	2011	32.0(21.5-44.7)	45.9(32.5-59.9)	1.6(0.4-6.4)	20.5(12.7-31.3)	38.9(29.8-48.8)
Sri Lanka	2015	19.7(6.8-45.0)	53.2(26.9-77.8)	—	27.2(11.4-51.9)	19.1(5.7-48.0)
Thailand	2015	67.1(58.5-74.6)	26.5(20.6-33.3)	—	6.4(2.9-13.7)	29.2(22.1-37.6)
Timor-Leste	2013	80.9(73.0-86.9)	16.1(10.9-23.2)	2.2(1.1-4.2)	0.8(0.3-2.6)	36.4(30.2-43.0)
**Western Pacific**						
Brunei Darussalam	2013	35.5(28.3-43.4)	50.2(41.3-59.2)	—	14.3(9.3-21.3)	43.0(32.1-54.6)
Cambodia	2016	36.1(20.8-54.9)	55.6(37.1-72.6)	8.4(1.8-31.1)	—	28.2(12.2-52.7)
Cook Islands	2016	21.2(11.3-31.1)	65.2(53.7-76.7)	—	13.6(5.3-21.9)	25.6(16.2-35.0)
Fiji	2016	55.1(48.1-61.9)	29.2(22.9-36.5)	—	15.7(12.3-19.9)	38.6(31.9-45.8)
Guam	2017	21.3(14.4-30.3)	49.7(40.0-59.4)	—	29.0(21.4-38.0)	24.7(17.9-33.0)
Kiribati	2018	61.9(55.8-67.7)	19.7(14.5-26.2)	—	18.4(13.8-24.1)	23.2(18.6-28.7)
Laos	2016	56.3(47.9-64.4)	39.7(32.0-48.1)	2.6(1.2-5.9)	1.3(0.4-4.0)	35.0(28.7-41.9)
Macao (China)	2015	60.5(46.3-73.1)	36.9(24.1-51.8)	—	2.7(0.4-16.9)	48.7(36.1-61.4)
Marshall Islands	2016	48.1(39.4-57.0)	31.8(24.5-40.1)	—	20.0(14.8-26.5)	25.8(19.9-32.8)
Micronesia	2013	42.3(38.8-45.8)	46.0(42.5-49.5)	1.7(0.8-2.6)	10.0(7.9-12.1)	32.4(29.3-35.5)
Mongolia	2014	71.4(63.3-78.3)	24.3(17.8-32.3)	—	4.3(2.1-8.5)	38.8(33.9-43.9)
New Caledonia	2010	32.6(23.6-43.2)	57.9(48.5-66.8)	—	9.5(5.6-15.6)	37.4(28.5-47.3)
Northern Mariana Islands	2014	29.3(21.6-38.4)	54.8(44.5-64.6)	0.5(0.1-3.4)	15.5(10.3-22.6)	24.3(18.2-31.5)
Palau	2017	17.1(13.0-22.2)	49.3(43.3-55.3)	—	33.6(28.2-39.4)	27.7(23.4-32.3)

Papua New Guinea	2016	74.0(67.6-79.6)	19.5(14.3-26.0)	—	6.5(3.7-11.0)	40.7(34.8-46.9)
Philippines	2015	81.3(77.2-84.8)	14.9(12.0-18.3)	0.6(0.2-1.5)	3.3(2.1-5.1)	36.8(33.0-40.6)
Samoa	2017	40.2(30.6-50.6)	47.5(35.7-59.6)	—	12.3(5.3-26.0)	24.7(15.6-36.6)
South Korea	2013	36.8(27.2-47.6)	50.0(40.3-59.7)	—	13.2(7.8-21.5)	15.0(10.4-21.3)
Tokelau	2014	4.7(1.3-15.4)	77.8(57.5-90.1)	—	17.5(6.5-39.4)	27.0(15.2-43.2)
Tonga	2010	29.4(21.4-38.9)	46.2(34.8-58.1)	3.0(1.6-5.5)	21.3(15.5-28.6)	41.2(35.5-47.3)
Tuvalu	2018	38.7(26.2-52.9)	39.1(27.9-51.6)	—	22.2(12.9-35.4)	8.3(4.1-16.2)
Vanuatu	2017	67.7(59.7-74.8)	0.5(0.1-3.6)	4.2(2.2-7.7)	27.6(21.0-35.4)	39.7(33.7-46.0)
Viet Nam	2014	70.0(57.2-80.3)	—	—	30.0(19.7-42.8)	64.2(49.0-77.0)

UNRWA, United Nations Relief and Works Agency.

†Data are presented as % (95% confidence interval).

— indicates data are unavailable.

Table S4. Proportions of cigarette purchase patterns (during the past 30 days) among current cigarette smokers by country, 2010-2018

Country/territory	Survey year	Pack†	Individual sticks†	Other patterns†
**Africa**				
Algeria	2013	34.1(25.9-43.5)	56.0(48.5-63.3)	9.9(6.1-15.5)
Angola	2010	100.0	0.0	0.0
Cameroon	2014	27.6(19.9-36.8)	48.0(32.1-64.4)	24.4(14.7-37.7)
Comoros	2015	38.1(30.7-46.2)	34.1(25.3-44.1)	27.8(17.7-40.8)
Gabon	2014	17.1(10.2-27.4)	69.9(58.8-79.1)	13.0(6.4-24.4)
Gambia	2017	34.9(28.1-42.4)	47.0(39.6-54.6)	18.0(12.1-26.0)
Ghana	2017	24.9(13.4-41.5)	46.9(23.7-71.5)	28.2(7.7-64.8)
Kenya	2013	20.5(11.8-33.0)	51.5(36.3-66.4)	28.1(17.7-41.4)
Madagascar	2018	3.9(1.2-11.6)	82.4(67.0-91.6)	13.7(5.7-29.5)
Mauritania	2018	26.0(17.9-36.2)	44.0(31.6-57.2)	30.0(21.9-39.5)
Mauritius	2016	18.7(11.1-29.8)	76.6(65.8-84.8)	4.7(2.7-7.8)
Mozambique	2013	40.0(22.4-60.6)	17.3(7.9-34.0)	42.7(22.4-65.8)
Senegal	2013	48.6(36.0-61.3)	23.6(8.3-51.5)	27.8(15.2-45.2)
Seychelles	2015	29.1(23.3-35.6)	56.0(48.7-63.1)	14.9(10.6-20.5)
Sierra Leone	2017	33.7(14.8-59.7)	54.2(28.6-77.7)	12.2(6.1-22.7)
Togo	2013	33.9(22.7-47.2)	49.8(39.6-60.1)	16.3(7.5-31.8)
Uganda	2018	16.3(4.7-43.6)	65.8(34.1-87.7)	17.9(6.0-42.6)
United Republic of Tanzania	2016	41.7(21.2-65.6)	33.4(12.0-64.8)	24.9(9.1-52.2)
Zimbabwe	2014	24.9(12.1-44.3)	13.9(7.3-24.9)	61.2(39.4-79.3)
**America**				
Antigua and Barbuda	2017	35.5(15.2-62.8)	40.2(19.2-65.6)	24.3(9.0-51.0)
Argentina	2018	73.9(49.4-89.1)	18.3(6.8-40.5)	7.9(3.1-18.8)
Bahamas	2013	28.2(11.9-53.1)	36.2(16.8-61.4)	35.7(16.4-61.0)
Barbados	2013	29.4(18.7-42.9)	24.8(17.1-34.6)	45.8(34.1-57.9)
Belize	2014	32.6(23.8-42.8)	47.4(35.4-59.8)	20.0(12.9-29.5)
Bolivia	2018	64.4(56.2-71.8)	32.7(25.6-40.8)	2.9(1.3-6.5)
Chile	2016	58.4(53.8-62.8)	34.5(30.2-39.0)	7.2(5.9-8.7)
Costa Rica	2013	44.4(31.3-58.3)	31.6(20.3-45.5)	24.1(15.2-35.8)
Cuba	2018	56.4(46.7-65.5)	38.8(29.8-48.6)	4.9(2.4-9.5)
Dominican Republic	2016	27.6(7.9-62.7)	33.6(12.3-64.7)	38.8(14.2-70.7)
Ecuador	2016	31.5(25.3-38.4)	58.1(52.1-63.9)	10.5(7.2-14.9)
El Salvador	2015	36.1(29.7-43.1)	48.7(41.8-55.7)	15.2(10.3-21.8)
Grenada	2016	29.9(17.6-46.0)	60.6(45.9-73.6)	9.5(4.7-18.2)
Guatemala	2015	26.5(21.9-31.7)	63.9(58.7-68.7)	9.7(6.6-13.9)
Guyana	2015	58.1(43.8-71.2)	19.5(12.2-29.7)	22.3(12.3-37.0)
Honduras	2016	32.3(22.1-44.4)	46.2(30.6-62.6)	21.5(10.6-38.8)
Jamaica	2017	23.7(15.2-32.2)	53.6(43.7-63.5)	22.7(14.4-31.0)
Nicaragua	2014	33.1(27.6-39.1)	58.3(51.8-64.5)	8.6(5.3-13.7)

Panama	2017	27.6(15.1-44.9)	63.6(45.1-78.8)	8.8(3.4-21.0)
Paraguay	2014	61.6(47.9-73.6)	18.9(10.2-32.4)	19.5(11.2-31.9)
Peru	2014	26.0(16.1-39.1)	38.3(24.2-54.6)	35.8(16.7-60.7)
Saint Lucia	2017	25.6(13.2-44.0)	40.0(28.0-53.4)	34.3(22.3-48.8)
Saint Vincent and the Grenadines	2018	40.6(21.2-63.4)	31.3(16.1-52.1)	28.1(13.1-50.2)
Suriname	2016	55.1(43.8-65.9)	35.8(26.4-46.5)	9.0(4.8-16.5)
Trinidad and Tobago	2017	45.2(36.4-54.2)	34.2(25.8-43.7)	20.7(12.1-33.0)
Uruguay	2014	52.2(44.2-60.1)	39.9(32.6-47.8)	7.8(4.6-13.1)
**Eastern Mediterranean**				
Afghanistan	2017	13.2(4.5-32.6)	58.9(39.5-75.9)	27.9(16.3-43.4)
Bahrain	2015	59.0(47.9-69.3)	11.0(7.0-16.9)	30.0(22.2-39.0)
Djibouti	2013	41.4(31.2-52.5)	42.6(31.7-54.2)	16.0(8.3-28.5)
Egypt	2014	29.6(11.2-58.4)	70.4(41.6-88.8)	0.0
Gaza Strip	2013	48.2(27.2-69.9)	34.9(20.1-53.3)	16.9(7.7-33.0)
Iraq	2014	62.5(55.7-68.8)	19.4(13.2-27.5)	18.2(10.6-29.5)
Jordan	2014	40.3(28.9-52.8)	43.7(34.8-53.1)	16.0(11.3-22.2)
Kuwait	2016	70.5(64.4-76.0)	6.2(3.4-11.1)	23.3(18.4-29.0)
Morocco	2016	38.3(23.2-56.1)	53.2(39.0-66.8)	8.5(1.3-38.8)
Oman	2016	59.5(42.5-74.6)	12.6(5.0-28.3)	27.8(13.8-48.1)
Pakistan	2013	27.2(12.2-49.9)	48.2(30.6-66.3)	24.6(11.4-45.4)
Qatar	2018	66.4(56.9-74.7)	19.5(12.5-29.0)	14.1(9.1-21.3)
Tunisia	2017	38.9(29.8-48.7)	50.7(41.0-60.5)	10.4(5.6-18.5)
United Arab Emirates	2013	62.5(53.3-70.9)	18.9(12.6-27.3)	18.6(13.0-25.9)
UNRWA GAZA	2013	26.5(17.0-38.8)	54.9(42.7-66.5)	18.6(12.3-27.1)
UNRWA Jordan	2014	33.4(20.5-49.3)	62.3(44.8-77.1)	4.3(1.9-9.5)
UNRWA Lebanon	2013	77.7(67.4-85.4)	8.6(4.1-17.0)	13.8(8.3-21.9)
UNRWA west bank	2014	38.1(27.9-49.5)	45.3(34.7-56.3)	16.6(10.9-24.5)
West BANK	2016	41.4(35.0-48.0)	37.9(29.5-47.1)	20.7(14.0-29.6)
Yemen	2014	24.4(8.9-51.5)	64.5(38.7-84.0)	11.0(3.9-27.7)
**Europe**				
Albania	2015	51.3(40.8-61.7)	40.3(29.5-52.2)	8.4(5.4-12.8)
Azerbaijan	2016	37.2(27.5-48.2)	46.7(32.8-61.2)	16.0(8.1-29.4)
Belarus	2015	88.0(80.3-93.0)	1.1(0.2-5.0)	10.8(6.0-18.7)
Bosnia and Herzegobina	2013	82.6(79.3-85.4)	4.4(3.0-6.4)	13.0(10.8-15.6)
Bulgaria	2015	68.0(62.2-73.3)	24.7(19.3-31.1)	7.2(5.2-10.0)
Croatia (Hrvatska)	2016	86.2(80.8-90.3)	5.8(3.6-9.1)	8.0(5.4-11.8)
Czech Republic	2016	68.0(60.9-74.3)	23.1(17.4-30.1)	8.9(5.8-13.6)
Georgia	2017	69.6(58.0-79.2)	22.8(15.0-33.1)	7.6(2.9-18.4)
Greece	2013	67.5(61.0-73.4)	3.4(1.8-6.6)	29.0(23.6-35.2)
Italy	2018	73.6(66.9-79.3)	9.0(5.8-13.9)	17.4(12.9-23.0)
Kazakhstan	2014	66.0(44.4-82.5)	9.2(2.0-33.4)	24.8(14.3-39.5)
Kosovo	2016	14.3(5.2-33.9)	23.0(13.8-35.8)	62.7(49.0-74.5)
Kyrgyzstan	2014	49.7(33.4-66.0)	38.7(24.3-55.5)	11.6(3.6-31.3)

Latvia	2014	84.4(79.6-88.2)	0.0	15.6(11.8-20.4)
Lithuania	2018	75.5(69.0-81.0)	12.1(8.2-17.7)	12.4(8.7-17.4)
Macedonia	2016	80.3(73.3-85.8)	9.4(6.0-14.3)	10.3(7.0-14.9)
Malta	2017	45.8(31.7-59.9)	33.3(20.0-46.6)	20.8(9.3-32.3)
Montenegro	2018	69.6(61.0-77.1)	13.6(7.7-23.0)	16.8(11.1-24.6)
Poland	2016	68.2(62.4-73.5)	22.3(17.5-28.0)	9.5(7.2-12.4)
Portugal	2013	69.6(60.0-77.8)	19.5(11.4-31.2)	10.9(7.8-15.0)
Republic of Moldova	2013	83.6(75.1-89.6)	8.1(4.5-14.2)	8.3(3.2-19.8)
Romania	2017	55.8(48.3-62.9)	40.4(33.8-47.3)	3.9(1.8-8.0)
Russian Federation	2015	79.3(70.9-85.8)	8.1(4.1-15.2)	12.7(7.2-21.4)
San Marino	2018	64.4(44.0-80.7)	0.0	35.6(19.3-56.0)
Serbia	2017	82.5(78.7-86.3)	10.0(7.0-13.0)	7.5(4.9-10.1)
Slovakia	2016	78.3(72.0-83.5)	15.6(11.3-21.1)	6.1(4.0-9.1)
Slovenia	2017	57.1(47.5-66.1)	19.0(12.7-27.5)	23.9(16.2-33.8)
Tajikistan	2014	0.0	52.4(17.9-84.8)	47.6(15.2-82.1)
Turkey	2017	63.5(61.7-65.3)	25.8(24.1-27.6)	10.7(9.7-11.7)
Ukraine	2017	74.4(62.6-83.4)	21.0(13.4-31.2)	4.7(1.8-11.6)
**South-East Asia**				
Bangladesh	2013	14.3(4.4-37.7)	85.7(62.3-95.6)	0.0
Bhutan	2013	30.1(22.6-38.8)	51.5(42.4-60.5)	18.4(12.7-26.1)
Indonesia	2014	24.4(18.9-30.9)	74.4(68.0-79.9)	1.3(0.7-2.3)
Myanmar	2016	29.6(23.5-36.5)	64.2(56.8-71.0)	6.2(4.0-9.5)
Sri Lanka	2015	9.0(1.1-46.0)	62.1(21.0-91.0)	28.8(4.6-77.4)
Thailand	2015	52.3(42.5-62.0)	21.6(15.6-29.1)	26.1(18.5-35.5)
Timor-Leste	2013	30.5(18.9-45.2)	45.5(35.8-55.6)	24.0(16.0-34.3)
**Western Pacific**				
Brunei Darussalam	2013	70.2(52.4-83.5)	16.3(8.8-28.1)	13.5(5.1-31.3)
Cambodia	2016	48.0(19.0-78.4)	37.5(12.4-71.8)	14.5(3.3-45.6)
Cook Islands	2016	42.9(26.5-59.3)	31.4(16.0-46.8)	25.7(11.2-40.2)
Fiji	2016	28.1(21.3-36.1)	55.6(48.8-62.2)	16.3(10.6-24.2)
Guam	2017	45.1(33.6-57.1)	32.7(23.6-43.3)	22.2(13.1-35.1)
Kiribati	2018	17.6(13.0-23.5)	72.7(65.7-78.7)	9.7(6.4-14.4)
Laos	2016	56.0(42.5-68.7)	39.6(27.7-52.9)	4.4(1.9-10.0)
Macau	2015	84.4(64.3-94.2)	7.5(2.3-21.8)	8.1(2.3-24.6)
Marshall Islands	2016	42.9(35.1-51.0)	45.0(37.3-53.0)	12.1(7.9-18.1)
Micronesia	2013	46.9(42.9-50.9)	33.4(29.6-37.2)	19.7(16.5-22.9)
Mongolia	2014	36.4(29.4-44.0)	59.5(51.9-66.7)	4.1(2.1-7.7)
Northern Mariana Islands	2014	63.9(54.5-72.3)	22.1(15.4-30.5)	14.1(8.7-21.9)
Palau	2017	51.4(44.2-58.5)	41.1(34.2-48.4)	7.5(4.4-12.6)
Papua New Guinea	2016	24.4(18.7-31.0)	55.8(47.4-64.0)	19.8(14.0-27.2)
Philippines	2015	14.7(10.6-20.2)	82.7(77.3-87.0)	2.6(1.6-4.2)
Samoa	2017	26.9(15.7-42.1)	58.1(45.6-69.7)	14.9(7.4-27.9)
South Korea	2013	82.9(75.9-88.2)	13.6(9.3-19.4)	3.5(1.4-8.8)
Tokelau	2014	79.1(62.3-89.7)	15.9(5.3-38.7)	5.0(1.0-22.4)

Tuvalu	2018	32.3(18.9-49.4)	49.0(33.3-64.9)	18.6(7.6-39.1)
Vanuatu	2017	23.0(16.6-31.0)	46.6(39.9-53.4)	30.4(23.2-38.7)
Viet Nam	2014	34.0(22.1-48.3)	56.5(43.0-69.0)	9.5(3.6-23.1)

UNRWA, United Nations Relief and Works Agency.

†Data are presented as %(95% confidence interval).
